# Current Demographics Suggest Future Energy Supplies Will Be Inadequate to Slow Human Population Growth

**DOI:** 10.1371/journal.pone.0013206

**Published:** 2010-10-05

**Authors:** John P. DeLong, Oskar Burger, Marcus J. Hamilton

**Affiliations:** 1 Department of Ecology and Evolutionary Biology, Yale University, New Haven, Connecticut, United States of America; 2 Department of Biology, Stanford University, Stanford, California, United States of America; 3 Departments of Biology and Anthropology, University of New Mexico, Albuquerque, New Mexico, United States of America; 4 Santa Fe Institute, Santa Fe, New Mexico, United States of America; University of Utah, United States of America

## Abstract

Influential demographic projections suggest that the global human population will stabilize at about 9–10 billion people by mid-century. These projections rest on two fundamental assumptions. The first is that the energy needed to fuel development and the associated decline in fertility will keep pace with energy demand far into the future. The second is that the demographic transition is irreversible such that once countries start down the path to lower fertility they cannot reverse to higher fertility. Both of these assumptions are problematic and may have an effect on population projections. Here we examine these assumptions explicitly. Specifically, given the theoretical and empirical relation between energy-use and population growth rates, we ask how the availability of energy is likely to affect population growth through 2050. Using a cross-country data set, we show that human population growth rates are negatively related to per-capita energy consumption, with zero growth occurring at ∼13 kW, suggesting that the global human population will stop growing only if individuals have access to this amount of power. Further, we find that current projected future energy supply rates are far below the supply needed to fuel a global demographic transition to zero growth, suggesting that the predicted leveling-off of the global population by mid-century is unlikely to occur, in the absence of a transition to an alternative energy source. Direct consideration of the energetic constraints underlying the demographic transition results in a qualitatively different population projection than produced when the energetic constraints are ignored. We suggest that energetic constraints be incorporated into future population projections.

## Introduction

Over human evolutionary history, the global human population has grown from an initial small size to ∼7 billion today. Recent global population growth rates peaked in the 1950's and 1960's but are now declining [1], and it is widely believed that the world's population size is approaching a steady-state. Demographic studies suggest that we can expect a leveling-off of the human population at about 9–10 billion by the middle of this century [2], [3]. Such projections are made by extrapolating recent trends in the relationship between time and vital rates. The key phenomenon invoked is the “demographic transition”, which is the reduction in fertility that follows the development-induced reduction in mortality [4], [5]. In essence, growth rates decline as a result of economic development, which brings benefits that increase lifespan and reduce infant mortality [6], [7]. Stimulated by these development benefits, fertility rates decline until they reach replacement levels or lower.

Economic development requires energy [7]–[11]. An expanded, energetic view of the demographic transition is that increasing energy use yields increasing development, thus decreasing mortality, and eventually decreasing fertility. Most projections have assumed that energetic inputs are either irrelevant for the demographic transition or that global energy supplies will be sufficient to fuel the economic growth that underlies the demographic transition [1], [3]. Such assumptions should be scrutinized for empirical reasons, but also because they contradict basic ecological theory. We submit that understanding the connection between energy and population growth in humans has the potential to shed light on the mechanisms of population regulation in the human species [12].

### Energy-dependent population growth

Energy is related to population growth via its effects on birth, death, and migration rates. When energy increases in supply, a population may grow [13], but energy supply may be approximately fixed over some time scales. This latter state is the basis for much classic theory in ecology, which suggests that as a population grows, per-capita access to energy declines, leading to declines in birth rates and increases in death rates and ultimately to a steady-state population size [14]. Indeed, recent studies show that metabolic rates (i.e., rates of energy use) are directly linked to birth and death rates [15]. The majority of biological populations experience some level of density-dependent population growth that is a function of intra-specific competition for available food energy [16], although other factors, such as predation and abiotic stressors, are also involved [17]. For our purposes here, we define an ecological path to zero growth as that in which individuals become energetically constrained to the point where birth rates equal death rates. This is the steady state that occurs in the classic logistic model of population growth, for example.

Importantly, however, modern humans use a considerable amount of energy in addition to that required to support their biological metabolism [18]. This extra-metabolic energy use has increased through time hand-in-hand with economic development [19]. Extra-metabolic energy is fundamentally different from the food energy that constrains the ecological path to zero growth, in terms of how it is acquired, the amounts that are involved, and the activities it fuels. Nonetheless, relaxing energetic constraints with the addition of extra-metabolic energy to the biological energy budget stimulates changes in energy allocation patterns, such that increased energy use results in fewer offspring. The use of extra-metabolic energy also increases survivorship [7], so it is possible that a high-energy steady-state could arise if there is an intersection of the curves relating energy use to fertility and energy use to mortality. The industrial path to zero growth is the trajectory in which the continued addition of extra-metabolic energy to the total energy use of individuals drives birth rates and death rates toward a steady state characterized by relatively large amounts of per-capita energy use [20].

The demographic prediction of a stabilized global population by mid-century rests on the ability of the global population to increase per-capita energy use and follow the industrial path to zero growth. Such a steady-state does not represent an ecological carrying capacity. It is a state where individuals with access to relatively large amounts of energy reproduce only at replacement levels. In this paper we ask whether future energy supplies are projected to be sufficient to allow the global population to follow the industrial path to zero growth. We quantify the empirical relationship between per-capita energy use and growth rate and use this relation to assess how future energy scenarios may affect the size of the human population through mid-century.

## Results

**Figure 1:**
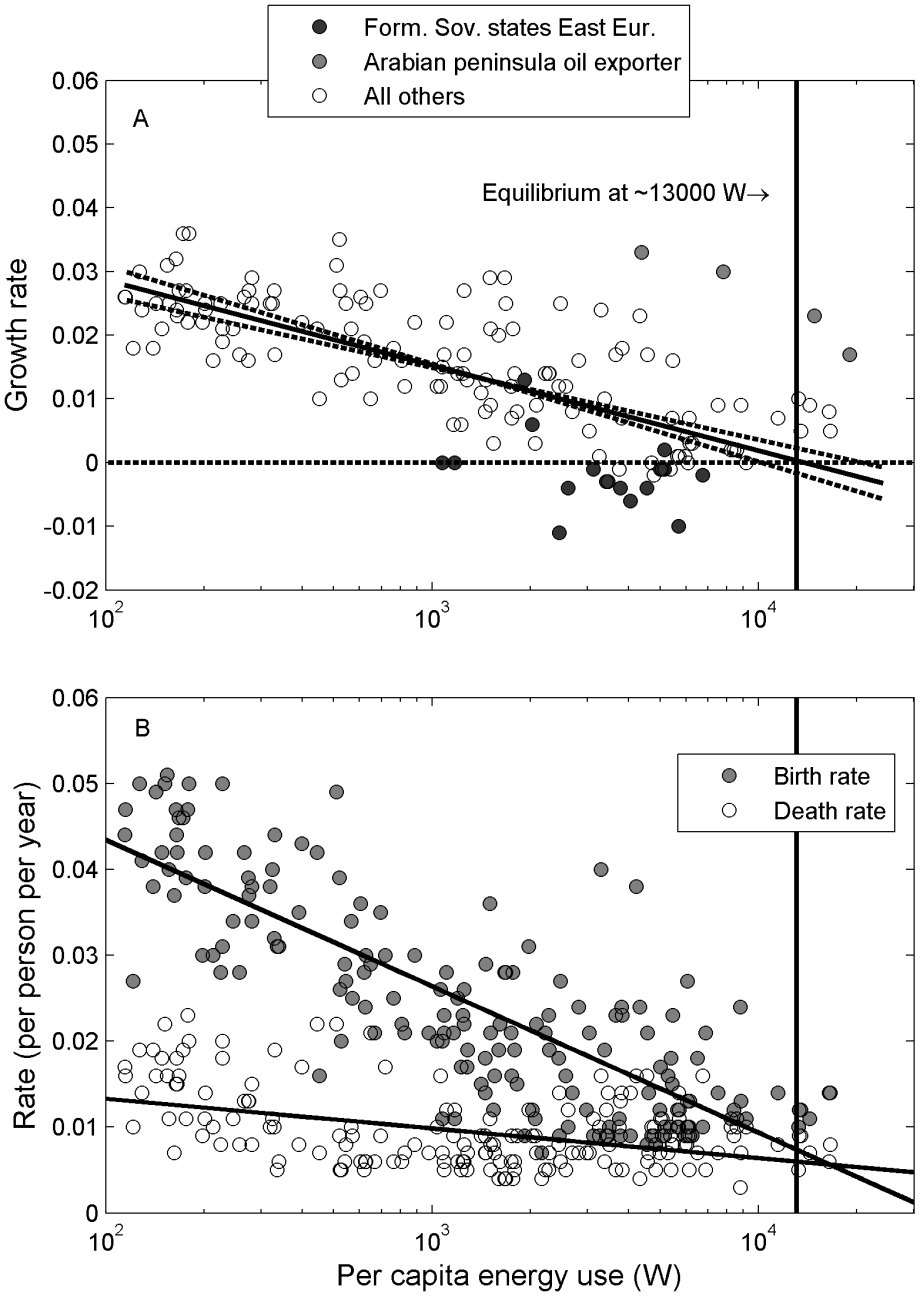
Relationship between demographic rates (2000–2005) and per-capita energy consumption for countries (2003). **A**. Growth rate declines across countries, with an equilibrium at ∼13,000 W. The solid line is a regression fit through the data (−0.0058 ln(*E*_pc_)+0.0553; R^2^ = 0.54), and the dashed lines are drawn with 95% confidence intervals of the parameter estimates. **B**. Both mean birth rates and mean death rates decline with per-capita energy consumption, but birth rates fall faster than death rates, generating the decline in growth rate observed in **A**. Fits are: birth rate (R^2^ = 0.67; equation is (−0.0074 ln(*E*_pc_)+0.0775); death rate (R^2^ = 0.19; equation is (−0.0015 ln(*E*_pc_)+0.0202).

The rate of population growth in humans is strongly and negatively tied to per-capita energy use (Figure 1A; R^2^ = 0.44). Growth rate is zero at 13,131 W (95% confidence intervals, 10,590–21,150 W), meaning that under current conditions the global population would stop growing if everyone had access to ∼13 kW of energy, or over 150 times more than basal metabolic rate [21]. A similar relationship is found for average energy use and average growth rate through time (R^2^ = 0.73). This pattern is a direct result of the dependence of development on energy use, and the dependence of fertility and mortality patterns on development. Growth rates decline with per-capita energy use because birth rates fall with per-capita energy use more quickly than death rates (Figure 1B). The decline in growth rate with per-capita energy use gives rise to an industrial path to zero-growth, as described above, where individuals with access to abundant energy have replacement fertility.

**Figure 2:**
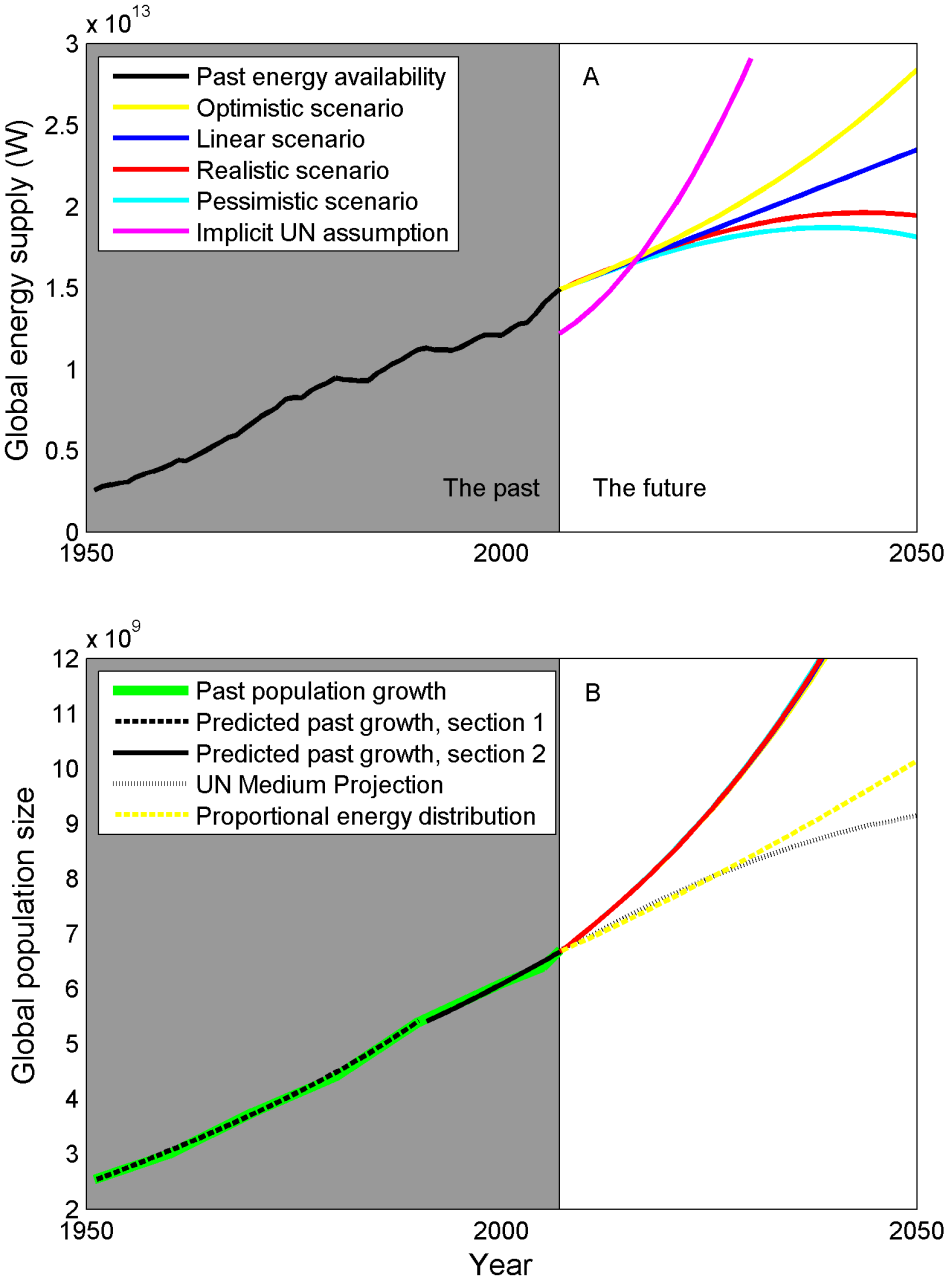
Past and future global energy availability and population size. **A**. Four scenarios bracket the uncertainty in future energy availability. A leveling of population at 9–10 b requires all people on the planet to have access to ∼13 kW on average, given current demographics. The quantity of energy needed to do this (pink line) is far outside any projection of future energy supplies. **B**. Projections of population growth given the four energy scenarios in **A**. All energy scenarios are insufficient to raise global per-capita energy supplies to the level at which growth rates reach zero, and therefore no stabilization of the population is seen. However, a lower trajectory (dashed yellow line) can be achieved by switching the distribution of energy use from the current state, where developed countries use 85% of total energy, to an egalitarian state, where the distribution is set by the proportion of people in the world by developmental status. Under this assumption, developed countries use 15% of the total energy supply. In this trajectory, the optimistic energy supply scenario is used.

Our model shows how variation in future energy supplies may affect the size of the future global population, given the empirical relationship shown in Figure 1. Specifically, our model suggests that future energy supplies will be insufficient to generate population leveling by mid-century (Figure 2A,B). We considered four alternative energetic projections, anchored by a concrete and recently published assessment of all types of primary energy [11]. These four scenarios, which we refer to as optimistic, linear, realistic, and pessimistic, all provide vastly insufficient amounts of energy to guide the global population down the industrial path to zero growth, given current empirical demographic data. The optimistic energy scenario is a continuation of the accelerating rate of energy use that has occurred over the last few decades, and exceeds the projected energy supply of any published account, yet even this amount of energy fails to bring global growth rates to zero.

In the four trajectories generated from the four energy scenarios, we hold the division of energy use between developed and developing countries constant at the current ratio of 85% to 15% of total energy supply used by developed and developing countries, respectively. We relaxed this assumption and allowed the distribution of energy to be proportional to population size, such that the developing world, with 82% of the world's population in 2009 [3], used 82% of the world's energy. Using the optimistic energy scenario, this change produced a much slower rate of growth, concurrent with the UN medium projection until about 2025, at 8 billion people, at which point our trajectory departs from the UN's and continues to rise.

How much energy is actually needed to allow the global population to follow the industrial path to zero growth? We calculated this amount, which we refer to as the implicit energy supply assumption of the UN medium projection, by breaking down the projection by developed and developing countries and determining the total energy supply needed to achieve the projected growth rate. Through about 2016, all energy scenarios suggest that there is an adequate global supply of energy to achieve the UN medium growth rate (Figure 2A, pink line). After this time, which is well before the predicted leveling of population size, the global supply falls short of the amount needed to continue along the UN medium projection.

**Figure 3:**
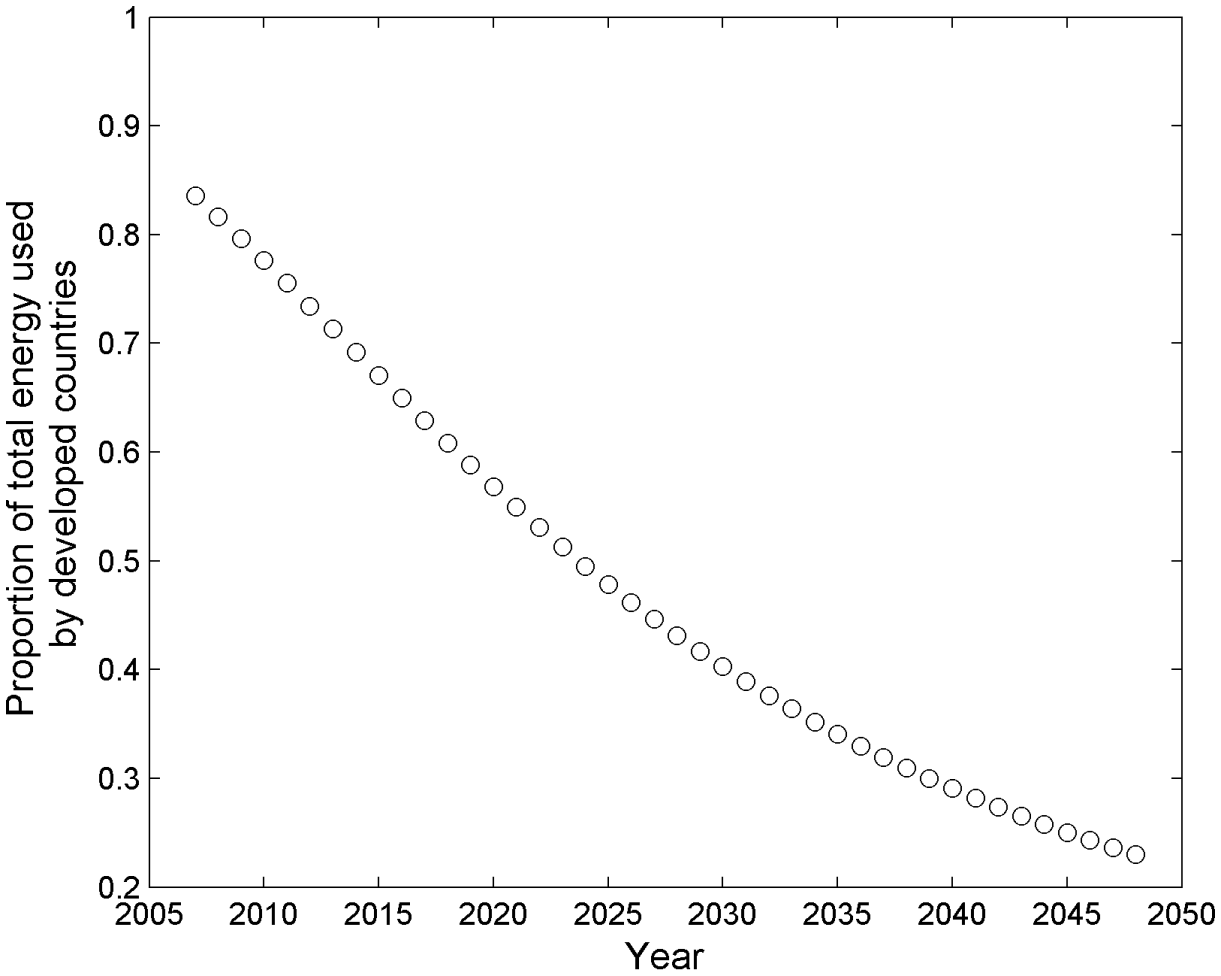
Following the UN medium projection requires not only large energetic inputs, but a shift in the distribution of the energy, from being dominated by developed countries (85%:15%) to being dominated by developing countries (25%:75%).

The implicit UN assumption suggests that there will be an increase in energy supply that appears to have no precedent in history [22]. Furthermore, the UN medium projection requires that the distribution of energy use between the developed and the developing world shifts through time, from a starting point of about 85% used in the developed world today, to nearly 75% used in the developing world by 2050 (Figure 3). Thus, the UN medium projection requires more energy than is predicted to be available, as well as for that energy to be distributed much differently than it is distributed today.

**Figure 4:**
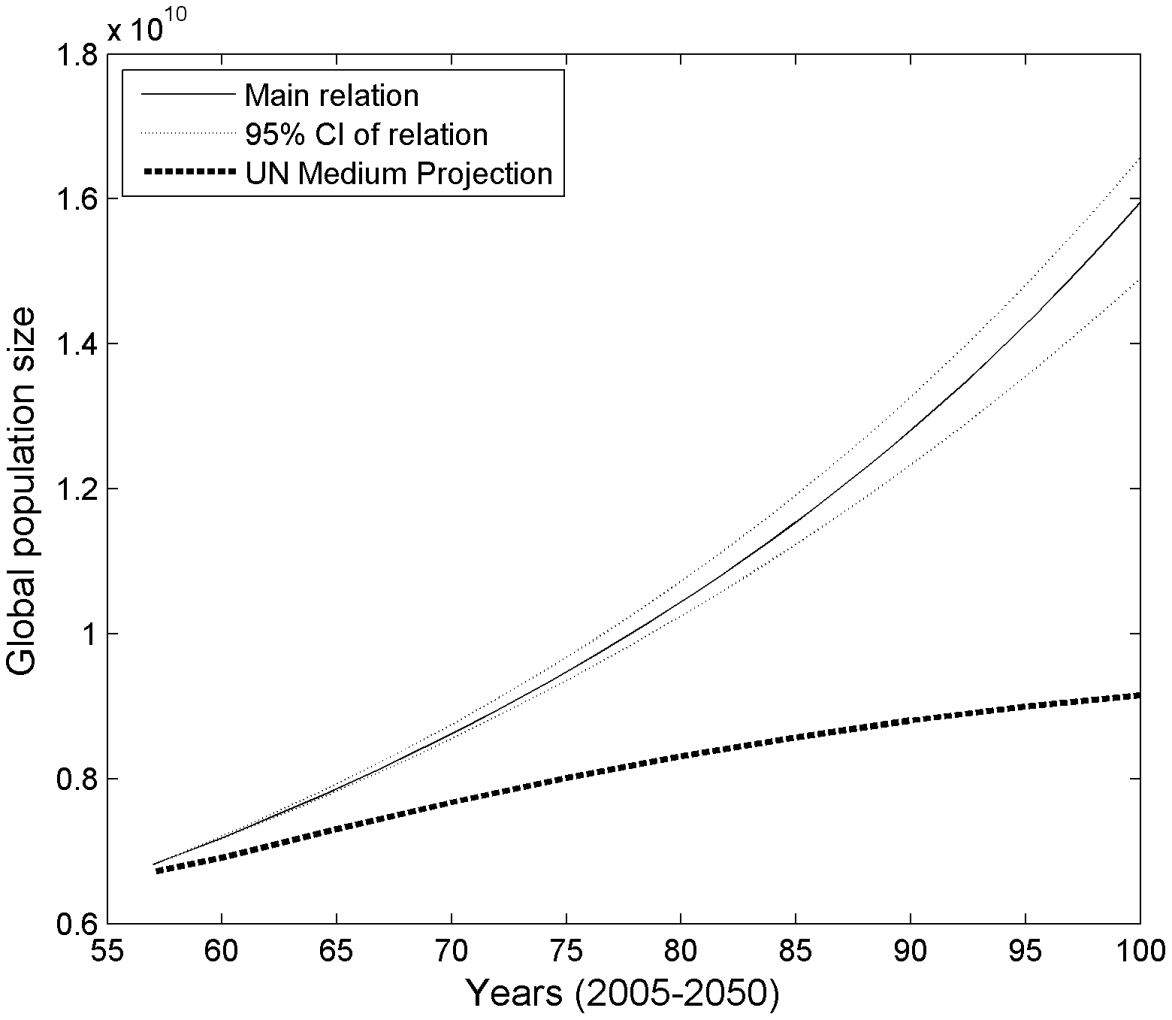
The effect of variation in the growth rate – *E*_pc_ relation on projected population growth. The main relation is the same as in Figure 1. Growth trajectories given for the optimistic energy scenario only, indicating that even with best-case energy availability, the error in the growth rate – per-capita energy use relation is not large enough to include a leveling to 9–10 billion by mid-century.

Of course, the outcomes produced by our model are sensitive to the precise relationship between per-capita energy use and growth rate, but Figure 4 shows that our main result is robust to error in estimating this relationship. Nevertheless, small changes in parameter values can cause the population trajectory to deviate from the observed historical trajectory, as a result of the compound nature of population growth involving billions of people. Matching growth from the model to the observed growth from 1950–1990 required a minor adjustment in the intercept of the model from 0.055 to 0.057 (Figure 2B, past growth, section 1). Matching growth from the model to the observed growth from 1990–2007 required a similar adjustment from 0.055 to 0.051 (Figure 2B, past growth, section 2). These adjustments to parameter *c* (see methods) are well within the 95% confidence intervals of 0.049–0.062.

**Figure 5:**
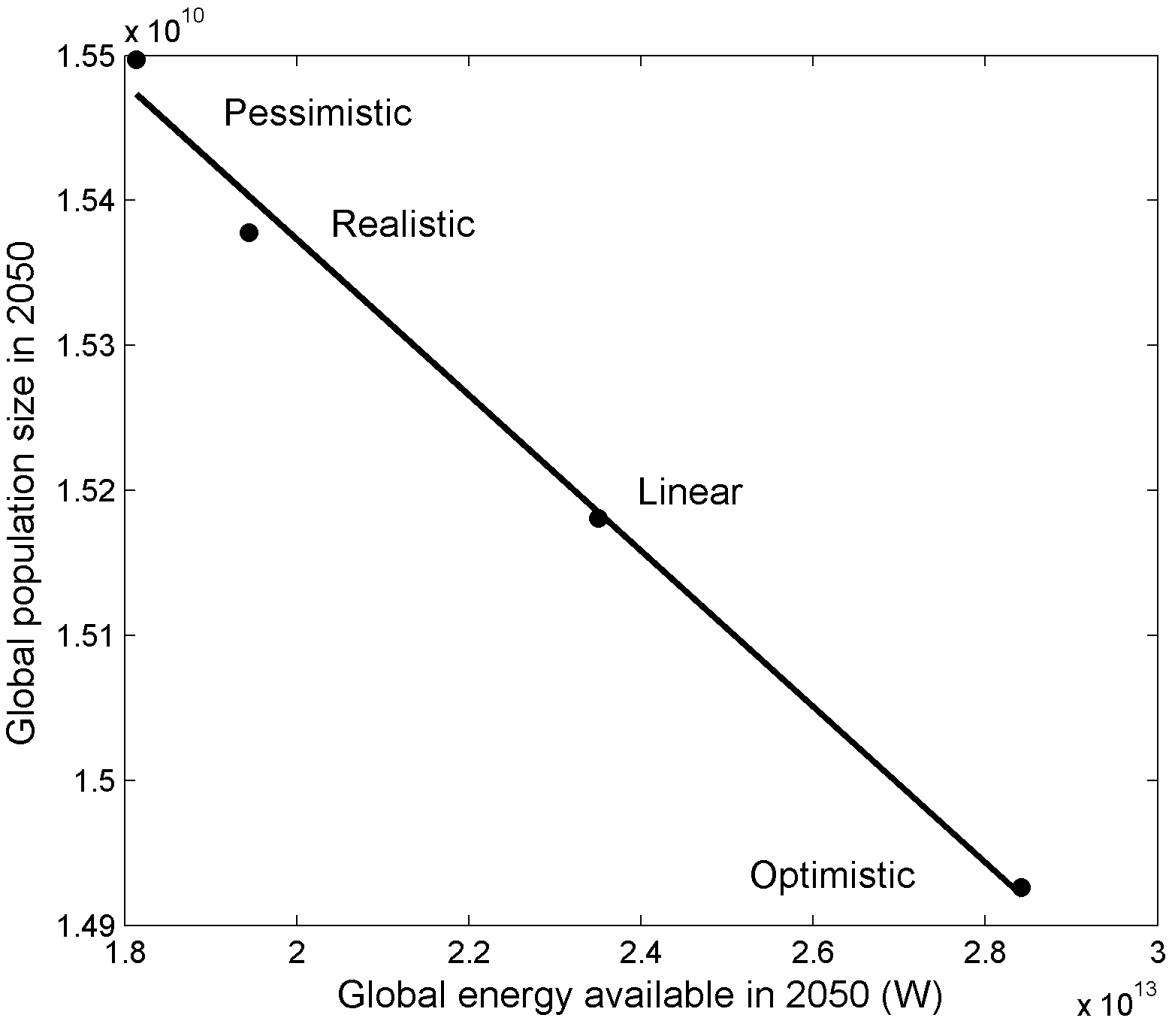
Inverse relationship between global energy supply in 2050 and global population size in 2050. This negative relationship is the direct result of a negative relationship between growth rate and per-capita energy use that generates an industrial, as opposed to an ecological, path to zero growth.

The negative relationship between energy use and growth rate also generates a negative relationship between global energy supplies and global population size at 2050, which is an unusual reversal of the typical positive relationship between resource supply and population size. This negative relationship is a unique signature of the industrial path to zero growth (Figure 5) and reinforces the idea that the industrial path to zero growth is one where additional energy leads to lower growth rates.

Finally, the model indicates that population growth will occur in both the developed and developing worlds, with the bulk occurring in the developing world because of asymmetric access to energy supplies (assuming that the developed world continues to use 85% of the world's energy supplies). This has consequences for the balance of per-capita energy use between the developed and the developing world. In 1950, per-capita energy use in the developed and developing world was 2600 W and 230 W, respectively (9-fold greater in the developed world). By 2007, the difference had grown to 7200 W and 470 W (15-fold greater), and by 2050, in the optimistic scenario, the imbalance is predicted to grow to 10,700 W and 470 W (22-fold greater). Continued population growth, coupled with the imbalance in energy use among developed and developing nations, sets the stage for continued growth through much of the world through the long-term suppression of per-capita energy use.

## Discussion

All attempts to project the future size of the global human population are subject to considerable, and unavoidable, uncertainty [23]. For most projections, the bulk of this uncertainty pertains to the rate and timing of the demographic transition. However, there is also much uncertainty in future energy supplies, and this energy is essential to fueling the economic development that results empirically in the demographic transition. It is therefore essential to understand how energy use patterns affect the growth and structure of the global human population [1]. By examining this relationship explicitly, we have identified a major, unrealistic assumption of previous population projections. Given the unequivocal relation between energy use and fertility, stabilizing the global population by mid-century will require vastly more energy than is currently projected to be available (Figure 2A, “Implicit UN assumption”). As the population grows, increasing amounts of energy are needed to bring more and more people to demographic equilibrium. Current average rates of energy use across the globe are much lower than equilibrium levels of ∼13 kW.

Demographic projections assume that the demographic transition is both inevitable and irreversible. We submit that both of these assumptions are problematic. This is because the demographic transition requires substantial amounts of energy, and if energy supplies decline, then growth rates will very likely rise. The potential for reversal of the demographic transition follows directly from conventional theory in population ecology and from the empirical relation we show here between energy consumption and fertility. In the event of such a reversal, growth rates will likely follow the current growth rate – energy relation. It is also possible that a new relation will emerge as time progresses [24].

The growth rate – per-capita energy use relation has developed through recent human history as human societies have increased access to finite pools of energy stored in the geosphere, namely fossil fuels [18]. Not surprisingly, then, there are interesting historical and cultural patterns embedded in this relationship. Some countries with shared histories cluster, including Arabian peninsula oil-exporters that have high growth rates for their energy use, and former Soviet states in Eastern Europe that have low growth rates for their energy use (Figure 1A). Some of this variation may have to do with recent migration patterns and world energy trade networks, and we suggest that further evaluation of historical effects on these relations may help us to better understand these patterns. We also point out that the variation around the general relationship indicates that there is scope for a reduction in the amount of energy needed to fuel the demographic transition.

The drop in growth rate with increasing per-capita energy use occurs because birth rates drop with energy use more quickly than death rates. How energy use induces a decline in death rates is fairly straightforward: energy is used to develop medical knowledge and technology and produce and distribute medical services [7], as well as support increased quantity and diversity of food that improves the nutritional state of people. In contrast, how the availability of extra-metabolic energy induces a change in birth rates is less clear. One possible explanation is that increases in the costs of raising children in more-developed countries forces the reduction in offspring number due to the constraint imposed by the time and energy available to allocate to total offspring number [5], [18], [25]. Currently, it is unclear why the introduction of extra-metabolic energy to the total energy budgets of industrial humans alters their reproductive allocation patterns, which in natural-fertility populations follow energy-based life history rules [26]. However, it is clear that understanding the energetic basis for reproductive decisions in humans could substantially contribute to our ability to affect future growth.

Today, it is widely assumed that the global human population will follow an industrial path to zero growth [2], [27], [28]. Our results suggest that the total quantity of energy will be insufficient to facilitate this outcome, given current demographics. Other constraints on birth and death rates may come into play at some point, but this would be very difficult to predict at this time. Shortages of water, disease, or violent conflicts could all play a role in limiting population size, or the population could become limited by food [29] and begin, again, to follow an ecological path to zero growth. Our analysis indicates that it is crucial to determine how those limits will come into play, as we can only expect the global human population to follow the industrial path to zero growth if future energy supplies turn out to be much greater than currently projected, and a greater balance among rich and poor nations in access to energy is achieved.

In conclusion, by failing to consider the fundamental theoretical and empirical relation between human reproduction and energy use, current demographic predictions of human population growth over the near future are at best questionable. Our analysis shows that by considering these relations as rigorously as possible, using empirical data and fundamental principles of ecological energetics, the global human population is likely to continue growing, due to energetic constraints that limit our ability follow the industrial path to zero growth under any reasonable prediction of future energy availability.

## Methods

### Data

We extracted data on per-capita energy use, growth rate, and crude birth and death rates from the World Resources Institute (WRI) database on country-level demographics and energy use [30], and we fit nonlinear models to the data to provide an empirical connection between energy and growth to use in the population model. Per-capita extra-metabolic energy use is defined as the total annual energy use for a country divided by the population size. The crude birth and death rate data from WRI are used only to show the intersection of birth rates and death rates at a unique value of per-capita energy use, illustrating why growth rates decline with per-capita energy use and how a high-energy zero-growth state exists for humans. Using the WRI data, we also tested for a relationship between per-capita energy use and growth rate through time, where growth rate was independently estimated as the average annual percent change of that country at mid-year. Data for Middle Eastern oil producing states were excluded as outliers, as they uniformly have far higher growth rates for their energy use, and this may be a function of the amount of energy used at a national scale in oil development.

We produced four scenarios of future energy supply, *E*, that bracket pessimistic to optimistic possibilities. For the “pessimistic” scenario we used an estimate of the future total primary energy supply from [11], which predicts continued growth of primary energy supplies followed by declines beginning after mid-century, and is consistent with other estimates [31]. We suspect, however, that this projection underestimates future renewable energy supplies, as the demand for alternative sources of energy will be quite large, spurring additional energy production. Therefore, the “realistic”, “linear”, and “optimistic” scenarios project larger future energy supplies than those predicted by [11]. The optimistic scenario represents increasing energy use consistent with recent decades. The four future energy scenarios are:
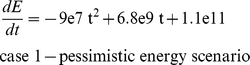


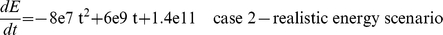


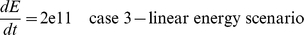


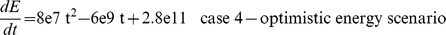



### Population model

In the model, changes in population size are given by *rN*, where growth rate *r* = *f*(*E*_pc_). *E* is given by the future energy scenarios, and *E*_pc_ is calculated as *E*/*N*. The function *f* is given by the empirically determined relationship in Figure 1A. The growth equation takes the form
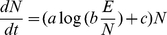
where *a* and *c* are fitted constants and *b* is the proportion of the global *E* available to a group. We divided the global population into the developed and the developing world because of the large imbalance in energy use between them (85% of global energy use in the developed world [30] and the large difference in population size (e.g., 5.32 billion in the developing and 1.35 billion in the developed in 2007 [30]. Thus, *b* is 0.85 in the developed world and 0.15 in the developing world. Global population sizes at year *t* are given as the sum of the developed and developing worlds.

We applied the model to past and future trajectories of global energy supply. Estimated energy use for the years 1950–2007 were provided by [11]. The four future energy scenarios for the years 2007 to 2050 were used to produce four energy-dependent future population trajectories. Initial conditions for 1950 were *E* = 2.5×10^12^ W [11], with population size in the developed world = 0.81 billion, and population size in the developing world = 1.72 billion [6]. The growth trajectories produced by the model were sensitive to the value of *c*, and conformity to past growth required slight alterations this parameter. The fitted value of *c* was 0.055 (+/−0.07), but a match to the growth pattern from 1950–1990 was achieved when *c* was set at 0.057. From 1990–2007, a value of 0.051 produced a match to the observed growth. For the years after 2007, the fitted value of *c* was used. To assess the robustness of our overall conclusion from the model output, we reran the model with the 95% confidence intervals for the parameters. We obtained three curves using the optimistic future energy scenario, which we show as providing a best-case scenario.

We calculated the future energy supply scenario implicitly assumed by the UN medium population growth trajectory using the following steps. We first broke the projection down into developed and developing worlds, given the endpoints of 2009 and 2050. We fitted a linear growth curve to the developed country growth curve and a quadratic function to the developing country growth curve. Sums of these two curves provided a close approximation to the UN medium projection. For each group, we calculated the growth rate for each year by (*N*_t+1_−*N*_t_)/*N*_t_ from the smoothed function. Third, we solved the fitted equation in Figure 1A for *E*_pc_, and used the calculated growth rate to estimate *E*_pc_ for each year. Finally, we multiplied *E*_pc_ for each year by *N*_t_ to produce *E*, the assumed global energy supply needed for the developed and developing nations to follow their respective curves, and then summed them to get the total global supply needed to propel the global population along the industrial path to zero growth. This procedure allowed us to compare the assumed global energy supply with the predicted energy scenarios, as well as to assess the assumed distribution of energy among developed and developing nations.
